# Physiology

**Published:** 1876-04

**Authors:** 


### Summary of Progress in the Medical Sciences.

### I. Physiology.

1. *The Urine of New-born Infants.* Parrot and Robin. (*Med. Times & Gazette*, London, Jan. 22, 1876.)

The following are the conclusions of a paper presented by MM. Parrot and Robin to the Académie des Sciences, (*Gaz. Hebd.*, January 14), "On the Normal Urine of New-born Infants, with Applications to Physiology and Clinical Medicine." 1. A new-born infant passes four times more urine than an adult, by kilogramme of its weight. 2. Under quite exceptional circumstances (as the urine of the first day, defective or faulty alimentation, etc.) the urine may furnish a very slight deposit, consisting of crystals of uric acid, of oxalate of lime, or urate of soda. Vegetable ferments seem to be more rapidly developed in it than in the urine of the adult. 3. It furnishes a neutral reaction with litmus. Acidity of the urine generally indicates that a too long interval has elapsed between the sucklings, and in some cases may point to a pathological condition. 4. The urine contains, on a mean, 3.3 grammes per litre of urea, or 80 centigrammes per kilogramme, in an infant of 3850 grammes; but an infant from 11 to 30 days old will render, in the twenty-four hours, about 90 centigrammes of urea, or 23 centigrammes per kilogramme of its weight. 5. The age, the weight and the temperature notably influence the quantity of urea. Before attributing differences that may be observed to a pathological condition, we must assure ourselves that the excess of urea surpasses the limits which we have assigned for variations due to these causes. 6. There exists a constant relation between the quantity of urea and the color and reaction of the urine, so that the inspection of these two characters enables us to estimate clinically the proportion of urea. 7. Traces of uric acid exist normally in the urine of the new-born, but they escape all dosage. The urine of the first day contains more. Extractive matters are not chemically appreciable, but the urine contains hippuric acid and allantoin. 8. Under no circumstances does the normal urine of the fœtus or the new-born contain albumen, and it exerts no reducing action on Barreswill's liquid. 9. The new-born consumes in the twenty-four hours, and per kilogramme of its weight, twice as much nitrogen as the adult; but it renders six times less by the urine, although it fixes at least as much oxygen. It therefore burns less, while absorbing more, of the combustible, and at least as much of the comburant. This excess of assimilation, demonstrated experimentally, is in harmony with the daily increase of weight—an increase which part of the oxygen absorbed contributes to. 10. When the urine becomes modified in any of its characters, beyond the limits traced, we should first direct attention to irregularity of alimentation, and then to morbid conditions. 11. Sometimes attention to the urine enables us to appreciate a special pathological condition, or a particular symptom, as œdema, diarrhœa, etc. 12. Such attention may sometimes enable us to foresee the near advent of accidents, such as œdema, athrepsia, etc., for lesions of nutrition precede the external signs of these affections ; and the child is already ill, although no symptom reveals its condition, of which the changes in the urine are the index.

